# Plant Transcription Factors Involved in Drought and Associated Stresses

**DOI:** 10.3390/ijms22115662

**Published:** 2021-05-26

**Authors:** Maria Hrmova, Syed Sarfraz Hussain

**Affiliations:** 1 School of Life Science, Huaiyin Normal University, Huai’an 223300, China; 2 School of Agriculture, Food and Wine, University of Adelaide, Glen Osmond, SA 5064, Australia; 3 School of Life Sciences, Forman Christian College (A Chartered University), Lahore 54600, Pakistan; sarfrazhussain@fccollege.edu.pk

**Keywords:** 3D structure, *cis*-elements, crops, plant biotechnology, stress-responsive mechanisms, transcriptional complexes, transcriptional regulation

## Abstract

Transcription factors (TFs) play a significant role in signal transduction networks spanning the perception of a stress signal and the expression of corresponding stress-responsive genes. TFs are multi-functional proteins that may simultaneously control numerous pathways during stresses in plants—this makes them powerful tools for the manipulation of regulatory and stress-responsive pathways. In recent years, the structure-function relationships of numerous plant TFs involved in drought and associated stresses have been defined, which prompted devising practical strategies for engineering plants with enhanced stress tolerance. Vast data have emerged on purposely basic leucine zipper (bZIP), WRKY, homeodomain-leucine zipper (HD-Zip), myeloblastoma (MYB), drought-response elements binding proteins/C-repeat binding factor (DREB/CBF), shine (SHN), and wax production-like (WXPL) TFs that reflect the understanding of their 3D structure and how the structure relates to function. Consequently, this information is useful in the tailored design of variant TFs that enhances our understanding of their functional states, such as oligomerization, post-translational modification patterns, protein-protein interactions, and their abilities to recognize downstream target DNA sequences. Here, we report on the progress of TFs based on their interaction pathway participation in stress-responsive networks, and pinpoint strategies and applications for crops and the impact of these strategies for improving plant stress tolerance.

## 1. Introduction

Drought is a major abiotic stress that severely affects crop productivity and distribution. Currently, up to 45% of the world’s agricultural land, where 38% of the world human population resides, is subjected to continuous or frequent drought [1,2]. Water is essential at every stage of plant growth from seed germination to plant maturation, and the shortage of water is the single most important factor that reduces global crop yields, with far-reaching socio-economic consequences [2]. Although under these circumstances, it is challenging to describe drought, in the agricultural context, drought is defined as a prolonged, abnormally dry period when the soil and atmospheric moisture are low, and the ambient air temperature is high, and available water resources are insufficient for agricultural needs [3]. Similarly, Lipiec et al. [4] noted that drought stress occurs when there is an imbalance between evapotranspiration flux and water intake from the soil. Drought with its associated complexity impacts agriculture and contributes heavily to the development of drought-prone areas leading to poor plant growth and reduces crop yields [5,6]. Hence, managing drought is about managing risks that associate with dry-land agriculture, aiming to reduce the impact of drought.

Considering that drought and related stresses and how they affect plants attract significant research, the understanding of plant protective mechanisms and how they are acquired, is of paramount importance. A large body of data related to drought was published specifically on transcription factors (TFs) and in this review we examine this contested topic in seven inter-related sections.

## 2. Drought Definition in the Agricultural Context

With the introduction of the ‘Food security and safety’ concept in 2002, The Food and Agriculture Organization of the United Nations (FAO UN) highlighted the global food scenario referring to ‘the situation when people, at all times, have physical, social and economic access to sufficient, safe and nutritious food’ [7]. The Intergovernmental Panel on Climate Change (IPCC) has warned the international community for action that climate change and global warming along with drought and associated stresses in agriculture are predicted to be worsened [8,9,10]. Hence, a comprehensive understanding of the impact of drought and associated stresses is critical for the development of climate-resilient crops that can adapt to changing climatic conditions [11].

Presently over 800 million people are globally undernourished [12], and over 50% yield losses in major crops have been recorded worldwide [2,13]. An additional 70% increase in crop productivity is required to feed around 10 billion people by 2050 [14]. Hence, the urgent demand for sustainable food production is far from realization due to the impact of drought and a variety of abiotic stresses [15,16,17,18] leading to lower incomes for farmers. For example, Daryanto et al. [19] reported as much as 40 and 21% reductions in maize and wheat yields, respectively, and similarly, Mariani and Ferrante [11] suggested the yield reduction of 34–68% in cowpea depending on the plant developmental stage when drought stress hits. Taken together, these adverse conditions are contributing to the increase of drought-prone areas around the globe with the loss of plant productivity [20,21]. These studies collectively provided evidence for the negative effects of drought stresses on crop yields singly or combined with other stresses [5,22].

## 3. Counter-Acting Drought and Associated Stresses through Crop Breeding, Gene Discovery, and Genetic Engineering

To adjust to environmental stresses, plants continuously adapt through dynamic adjustments at morphological, physiological, and biochemical levels during mainly their vegetative and reproductive growth stages, enabling them to survive harsh conditions [23,24]. Goufo et al. [25] listed several morphological and physiological adjustments, which offer a plant a relief from stress conditions, including the development of deep root systems, deep and sunken stomata, and stomatal closure to reduce their conductance, a decreased leaf area by leaf rolling or folding, and an increased leaf thickness to lessen evapotranspiration [25]. Similarly, the enhanced production of cuticular wax is an adaptive response to reduce water loss through evapotranspiration [26,27]. As for plant tissues, the reproductive stages of plants are critically prone to damage by heat and drought, since these stresses affect fertilization, and early seed developmental stages, leading to a complete crop failure or a decrease in seed number and sizes and the overall seed quality. Stress tolerance strategies in plants tend to maintain tissue hydrostatic pressure, by physiological and biochemical modifications, mainly through osmotic adjustments [28,29]. However, such strategies that plants have developed during billions of years of evolution are not well suited to cope with complex abiotic stresses in most crop plants [6,13].

Conventional plant breeding has contributed to developing abiotic stress-tolerant crop plants, however, there is a necessity for alternatives tools due to the complexity associated with tolerance traits, as these are controlled by multiple genes present at multiple quantitative trait loci (QTL) [30]. Landraces and wild grass relatives are known to have high abiotic stress tolerance and these desired traits can be crossed into crop species from wild grasses. Up to an estimated 10–20% of the wild variation traits were introgressed into modern wheat varieties [13]. Despite challenges, there are noteworthy examples of improved heat and drought tolerance of crop plants through conventional plant breeding efforts. Ripper, a drought-tolerant wheat variety was developed which performs well with high grain yields and enhanced milling and bread-making characteristics under non-irrigated conditions [31]. Similarly, Badu-Apraku and Yallou [32] developed maize varieties that performed well with high yields compared to control maize under both drought and biotic stresses. Three other groups independently demonstrated promising results by involving QTL markers in breeding programs in combination with marker-assisted backcrossing and marker-assisted recurrent selection strategies [33,34]. Following this breeding-based strategy, researchers introgressed QTLs from wild emmer wheat to improve drought resistance in elite durum (*Triticum. turgidum* ssp. *dicoccoides*.) and bread (*Triticum aestivum* L.) wheat cultivars through a marker-assisted selection [33]. Another study mapped QTLs under a combination of drought and heat stresses using a recombinant inbred line population [34]. These authors found that markers could be used as potential candidates for the identification of superior allelic variations in wheat populations [34]. 

The alternative to classical breeding is combining high throughput emerging technologies to discover and characterize key loci controlling plant stress tolerance [35]. Here, molecular biology techniques in combination with robust DNA sequencing, bioinformatics, and genetic engineering platforms could revolutionize plant biotechnological research [2,14,23,36,37,38,39].

## 4. Plant Stress-Responsive Mechanisms during Drought and Other Abiotic Stresses

Understanding the stress-responsive mechanisms that damage plants during abiotic stresses is critical for the breeding of tolerant and resistant crop plants. Plants have evolved various mechanisms to cope with stresses, and these include the shifts in the physiology of the plant and the expression of stress-associated genes, leading to the formation of a wide variety of low molecular mass metabolites collectively known as compatible solutes [2]. These solutes play a significant role in stress tolerance by preventing detrimental changes at cellular levels and the accumulation of these molecules actively or passively helps plants retain water and maintain turgor, protect protein structures, and stabilize cellular membranes under drought [2,40,41,42,43,44,45]. Among these osmolytes, proline is used in transgene-based research and is the main contributor to osmotic adjustments [46], although the molecular and cellular function of this solute is not entirely clear.

Costa et al. [41] showed that abscisic acid-insensitive 5 (ABI5) proteins are potential regulators of the expression of the late embryogenesis abundant 4 (LEA) proteins, which may be important factors in the longevity of *Xerophyta viscosa* in a desiccated state. Another example involves two groups of proteins involved in *Arabidopsis thaliana*—the first group is directly involved in plant protection, and the second group serves in signaling cascades and during transcriptional control [42].

**Figure 1:**
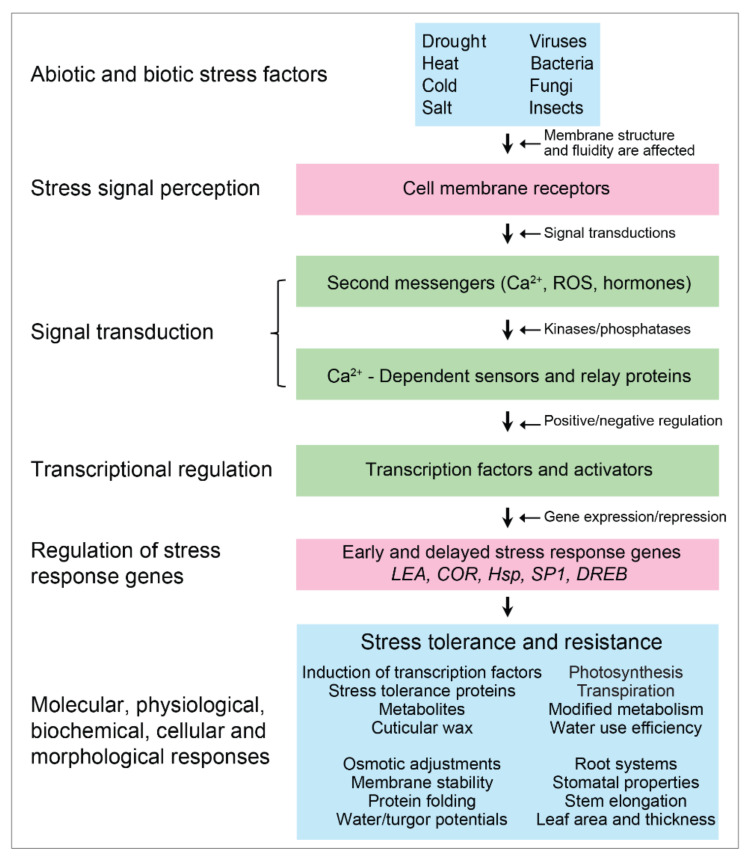
Plants respond to abiotic and biotic stresses through signal transduction pathways. An extracellular signal is perceived via receptors localized in membranes that lead to the activation of signal transduction pathways. The signal cascade (through second messengers, sensors, and kinases that activate a variety of TFs) results in the expression of multiple stress-responsive genes, which mediate stress tolerance and resistance, and restore the cellular and tissue homeostasis [6,18,25].

Key players in the first group of stress-responsive proteins include LEA proteins, chaperones, osmotins, anti-freeze proteins, mRNA binding proteins, enzymes involved in osmolyte biosynthesis, water channels and transporters, sugar and proline transport proteins, detoxification enzymes, and a variety of proteases. From this first group, LEA proteins are particularly interesting as they represent ‘the continuing conundrum of function’ [2]. The LEA proteins attribute their function in part to their structural plasticity or flexibility, as they are largely lacking secondary structures in the fully hydrated state but can fold during water stress and/or through association with membrane surfaces. Useful examples of LEA proteins are dehydrins, which prevent the coagulation and inactivation of proteins during water deficit through the protein anti-aggregation mechanism. The activation of signal transduction pathways triggered by abiotic (drought, heat, salt, cold) and biotic (viruses, bacteria, fungi, insects) stresses is linked to their progression from the perception of stress to plant adaptive stress response (Figure 1). In an effort for plants to survive and fulfill the criteria for natural selection, the evolution originates at the DNA level, ramifying one gene into two, etc. to eventual speciation [43,44]. This leads to the continuous development of plant adaptation to environmental adversities at molecular, biochemical, physiological, and morphological levels.

After plants are exposed to stress, the stress signal is received on the cell surface receptor, whereby signal transduction occurs through the accumulation or release of Ca^2+^ ions, and/or through the de novo synthesis of the second messenger molecules such as reactive oxygen species (ROS), inositol trisphosphates, hexaphosphate, and diacylglycerols [47]. These second messengers start regulating intracellular concentrations of cytosolic Ca^2+^ ion levels that lead to the biosynthesis of Ca^2+^ ion-binding proteins which activate calcium-dependent protein kinases (CDPKs) [48] and other relay proteins [44]. In turn, activated kinases and phosphatases initiate phosphorylation/de-phosphorylation of specific TFs, leading to the expression of stress-responsive genes [6]. As a result, switching on a series of phosphorylation/de-phosphorylation cascades via presumable kinase and phosphatase enzymes leads to stress tolerance. During this stress tolerance phase, the expression of functional genes involved in cellular protection in vegetative tissues results in the accumulation of various stress resistance proteins and molecules (Figure 1), and as a result, plants surpass unfavourable conditions [6].

## 5. Transcriptional Regulation of Plant Responses to Drought and Associated Stresses

Plants have evolved defense strategies to environmental stresses including interconnected networks at the molecular level which are controlled by complex signal transduction cascades. These strategies have three components: (i) Signal perception, (ii) signal transduction, and (iii) stress-responsive gene expression. The first step in the activation of a signal cascade is signal perception through cell-surface receptors that detect a stress stimulus [49]. Specific receptors perceive and recognize different signals and stimuli from the environment. The receptor-like kinase (RKL) was the first receptor kinase protein described in plants and since this finding, extensive efforts have been devoted to discovering other specific receptor kinase genes involved in plant responses to a variety of abiotic stresses [50,51,52]. Stress perception is followed by the activation and transduction of the external signal to internal signals in systemic signaling cascades. Secondary messengers such as ROS, Ca^2+^ ions, and phytohormones including ethylene (ET), abscisic acid (ABA), salicylic acid (SA), and jasmonic acid (JA) coordinate signal transduction pathways during stress response [53,54]. Major signal cascades and protein kinases and phosphatases involved in these cascades that are activated in plants after stress perception are the mitogen-activated protein kinase (MAPK) and the calcium-dependent protein kinase (CDPK), calcineurin-B-like proteins (CBLs), the CBL-interacting protein kinase (CIPK), and the protein phosphatase 2Cs (PP2Cs). The uncovering mode of action and the regulation of signal transduction pathways and manipulation of components of these pathways have fundamental importance for the genetic engineering of drought-tolerant plants.

### 5.1. Signal Transduction Pathways and Their Regulation by Plant Hormones

Plant growth and development are controlled by phytohormones released by plants. One such hormone is ABA, which helps in governing the development and growth of plants [55,56,57,58,59,60]. ABA is also regarded as a ‘stress hormone’ as it mediates adaptive responses of plants to abiotic and biotic stresses [61], and numerous reports characterized the involvement of TFs in a range of stresses that are perceived through pathways that up- or downregulated TFs (Figure 1). Nemhauser et al. [62] characterized simultaneously over 200 TFs belonging to 20 gene families using positive or negative regulation by ABA at a single developmental stage, although molecular mechanisms of individual TFs are poorly understood. Many drought-responsive genes are involved in ABA-signaling pathways, although reports indicated that some drought-induced genes show no response to ABA-signaling [63]. Based on these observations, the pathways leading to plant adaptation to abiotic stresses are divided into ABA-dependent and ABA-independent pathways [42,64].

The adaptive response of plants to drought and related stresses proceeds as follows. As water becomes depleted under water deficit, a rapid change in the hormonal level in plants is noticed including a rise in leaf ABA and/or a decline in cytokinins. The multifaceted response of leaf ABA levels leads to cell wall extensibility and, in some plants, to changes in roots hydraulic conductance, and tissue turgor [65]. Various genes involved in ABA biosynthesis and biosynthetic pathways in a range of organisms including plants were elucidated [61]. In addition to plants, ABA is synthesized by plant pathogenic fungi using two distinct pathways which are different from plant ABA biosynthetic pathways [66,67]. Cutler et al. [55] with ABA analogs suggested that ABA may have both intracellular and extracellular sites of perception. Proteins sensing ABA include ABA-binding proteins from barley aleurone, bean epidermal proteins, and a variety of G protein-coupled and other receptors isolated from plants. When investigating the *Arabidopsis thaliana* ABA-binding Pyrabactin Resistance (PYR) receptor proteins through a yeast two-hybrid system, the C2 class of PP2C protein phosphatases acted as negative regulators of ABA signaling. The mechanism of binding of ABA to the PYR receptors and PP2C phosphatases that dock to ABA-bound PYR receptors were elucidated at atomic levels [68].

Plants under environmental stresses, especially drought, exhibit modified growth through the coordinated action of several plant hormones, proteins, and regulatory factors and by inducing various responses, such as changes in transcription and post-transcriptional processes and the modulated expression of stress-responsive genes [55]. It has been estimated that in *Arabidopsis* around 5–10% of ABA-regulated transcriptional changes were affected. Approximately half (50%) of these changes were induced by exogenous ABA treatment, drought, and salinity including various TFs, protein kinases and phosphatases, dehydrins, transport proteins, and ROS detoxifying enzymes. Some of ABA repressed genes such as ribosomal, plasma membrane, chloroplast, and structural cell-wall genes were directly linked to plant growth and protection (Figure 1). Similar reports revealed the high frequency of unannotated transcriptional products regulated by ABA, which may represent intergenic regions [69]. These data indicated that a significant gap exists in our understanding of ABA-regulated signal transduction pathways under drought and associated stress. 

**Figure 2:**
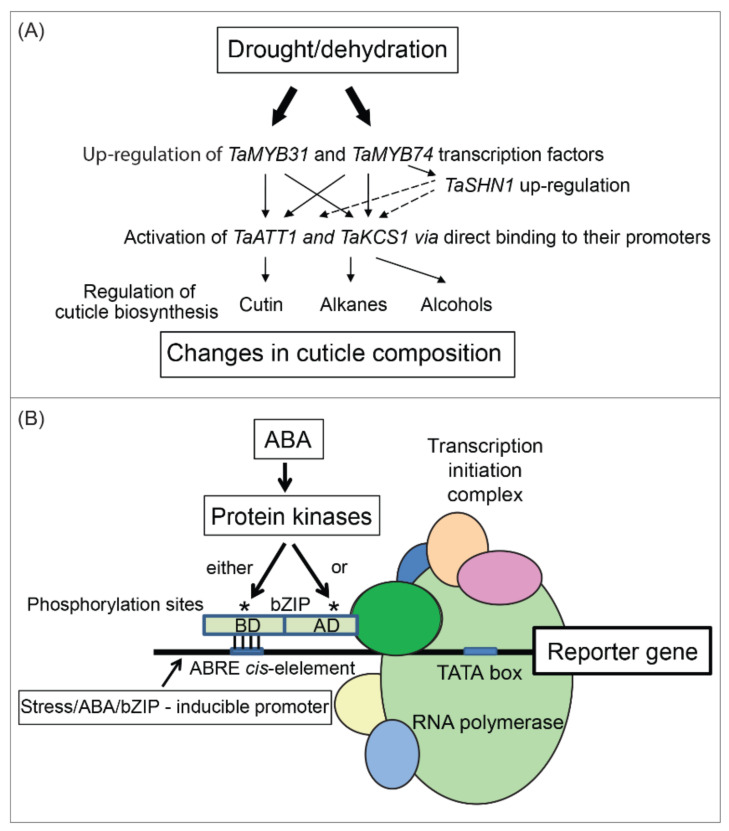
Regulatory mechanisms of wheat stress-responsive genes involved in drought and related stresses. (**A**) The proposed roles of wheat (*Triticum aestivum* L.) TaWXPL1D and TaWXPL2B, and TaMYB31 and TaMYB74 TFs in the regulation of cuticle biosynthesis during a wheat response to drought stress (modified from [26,27]). (**B**) Schematics of the likely mechanism of activation of bZIP TFs through post-translational modifications. This experimental design based on [84,85] is explained in the text.

Based on the current data, TFs involved in ABA-regulated pathways could be the natural targets for genetic manipulation to breed abiotic stress-tolerant crop plants. For instance, the overexpression of ABA pathway-related TFs resulted in an ABA-hypersensitive phenotype with enhanced osmotic stress tolerance in transgenic plants [70,71]. Transgenic plants overexpressing root architecture-related TFs showed high drought tolerance by promoting root growth and enhanced water use efficiency [72]. Several other potential TFs [26,27] such as those stimulating wax deposition in the cuticle and suberin deposition [73], and other regulators modulated ABA-regulated pathways (Figure 2). These TFs and regulators could potentially be used to activate stress-related genes leading to enhanced plant stress tolerance [26,27,74,75].

### 5.2. Transcription Factors and Their Key Regulatory Roles

TFs are central regulators of changes in gene expression and have fundamental importance in critical aspects of plant function, including the development of plants under stress and in ensuring that plant growth and development match fluctuations in environmental conditions [6,76,77]. The central role of TFs is in their ability to bind specific DNA sequences, make connections with different proteins secondary structures of transcriptional complexes, and play a synergistic role in the regulation and expression of almost any gene [6,14,78]. As noted previously, TFs are proteins that bind to local or distal *cis*-elements of a specific gene and control the flow of genetic information. *Cis*-elements or *cis*-motifs are defined as the conserved nucleotide sequences that are present upstream of promoter regions of genes and that mediate gene regulation by providing sites for TF binding. Research has highlighted mechanistic engagements of TFs on their interactions, local DNA structure, and genomic features and how this affects TF binding to specific DNA sequence motifs [79]. Notably, fully sequenced genomes of several plants showed that around 10% of all identified genes encode TFs [18]. A total of 1922, 1611, 2450, and 3,337 TFs were reported in *Arabidopsis thaliana*, rice, sorghum, and maize, respectively [18,80]. Wang et al. [80] classified known TFs in up to 60 families, based on their primary sequences, 3D structures of binding domains, DNA binding motifs, oligomerization patterns, and post-translational modifications.

### 5.3. Post-Translational Modifications and Protein-Protein Interactions of Transcription Factors

One of the most important post-translational modification in living organisms is phosphorylation/de-phosphorylation that occurs through a range of kinases and phosphorylates. An example includes the sucrose non-fermenting-1-related protein kinase 2 (SnRK2) that belongs to the family of plant-specific Ser/Thr kinases. In the absence of ABA SnRK2 remains in an inactive non-phosphorylated state by PP2C, resulting in the blockage of downstream phosphorylation cascades. The presence of ABA keeps SnRK2 in an active phosphorylated state, and thus ABA acts as a positive regulator of ABA-mediated signaling pathways [81]. It was demonstrated that phosphorylated SnRK2 plays a key role in the activation of mitogen-activated protein (MAP) kinase (MAPK) pathways (MAP3K-MAP2K-MAPK) or that these kinases activate the Ca^2+^-dependent protein kinase (CDPK) or the casein kinase II (CKII), which in turn phosphorylate target TFs, e.g., basic leucine zipper (bZIP) TFs. Several reports showed direct physical interactions between SnRKs and bZIP TFs, and direct activation of bZIP TFs by SnRK2s [82,83]. Therefore, it is not unexpected that specific classes of TFs in functional conformational states need to be engaged under different biological contexts including drought and other stresses.

The bZIP family is one of the largest and most diverse TF families [84,85] and the functional importance of bZIP TFs in abiotic (drought, salt, cold stresses) and biotic (pathogen defense) stresses through ABA signaling was documented [84,85]. The bZIP TFs are also involved in various plant physiological and developmental processes [86,87]. It is known that post-translational modifications such as phosphorylations/de-phosphorylation events are the main mechanism of bZIP TFs regulation (Figure 2), although this regulation mechanism is not fully understood. Fuji et al. [88] reported that the *Arabidopsis thaliana* Snf1-related protein kinase (SnRK2) phosphorylated the members of the ABA-binding factor clade of bZIP TFs. Yoshida et al. [89] demonstrated that four *Arabidopsis thaliana* bZIP TFs (ABF1, AREB1/ABF2, ABF3, and AREB2/ABF4) as downstream genes of the ABA-dependent drought signaling pathway were targets of SnRK2s [90]. The triple mutant analysis using AREB1, AREB2, and ABF3, suggested SnRK2s as the master regulators of drought responses in *Arabidopsis thaliana* [91]. This finding is significant since at least one class of physiologically relevant targets of SnRK2 kinases are bZIP TFs that are engaged in gene activation in response to ABA [55,56,88]. These reports also revealed kinase involvements in stress response in transgenic plants [73,85,92,93]. These findings collectively indicate that bZIP TFs could serve as significant targets for the genetic engineering of drought tolerance in plants. An example of testing the significance of phosphorylation sites is included in Figure 2B. Here, in silico analyses allow investigations of individual phosphorylation sites on bZIP TFs through side-directed mutagenesis. Variants are tested to find out if phosphorylatable residues mediate contacts with *cis*-elements through binding domains (BD) using Electrophoretic Mobility Shift or yeast one-hybrid assays. An obvious choice is to substitute Ser, Thr or Tyr residues with negatively charged phosphomimetics. These phosphorylation sites could also be situated on activation domains (AD), and when their phosphorylation did not affect DNA binding properties of bZIP TFs, then the effects of mutations could be tested via transient expression assays in cell cultures [84,85].

## 6. Molecular Structure and Regulation of Transcription Factors during Drought and Associated Stresses

Vast data are available on TFs and their roles in transcriptional regulation of stress-responsive genes in plants under abiotic stresses. In the first sub-section, we focus on the regulation of bZIP, homeodomain-leucine zipper (HD-Zip), and WRKY TFs [18,60,76,94], while in other sub-sections we detail the information on the molecular structure and properties of selected TFs involved in drought. 

The expression profiling of bZIP, HD-Zip, and WRKY under a variety of abiotic stresses in plant species highlighted that these TFs are involved in ABA-regulated developmental networks, addressing the abiotic stresses positively or negatively to pause the genetically pre-programmed growth and development, and consequently provide protection [6,76,94,95,96]. Ali et al. [97] in soybean identified GmbZIP110 through digital gene expression profiling of two cultivars with contrasting drought tolerance traits under drought and salinity. The functional characterization of GmbZIP110 in plants revealed that this TF could positively influence the expression of several downstream stress-responsive genes leading to the high accumulation of proline, which confers stress tolerance [97]. Relatedly, Hartmann et al. [96] studied the functional regulation bZIP1 and bZIP53 in *Arabidopsis thaliana* salt-treated roots and demonstrated re-programming roles of these TFs in root metabolism and adaptation to salt stress.

**Figure 3:**
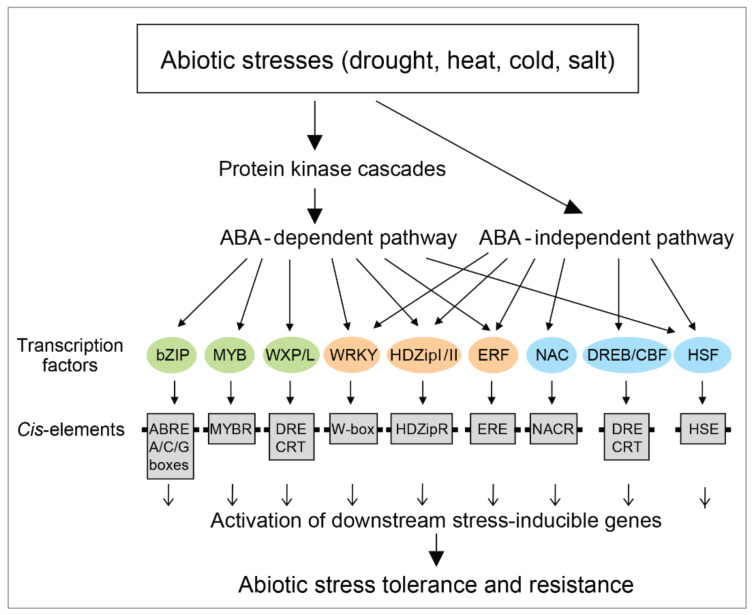
Biotic and biotic stresses are perceived by plants through ABA-dependent and ABA-independent pathways or between cross-talks of these pathways (based on [26,27,84]). Color coding differentiates between TFs that are controlled by ABA-dependent (green), ABA-independent (cyan) and ABA-dependent/independent (orange) pathways.

Under the water deficit, several TFs including bZIP, HD-Zip, and WRKY TFs are transcriptionally regulated through the ABA-dependent pathway (Figure 3). These TFs play key roles in ABA transduction pathways and regulate stress-responsive genes that are involved in the protection of cell integrity and plant development under stresses including drought and salinity stresses (Figure 2 and Figure 3). Despite extensive research, limited data and the precise functional information on developmental and stress-responsive bZIP, HD-Zip, and WRKY TF networks and cascades are available [98]. This leads us to further explore their roles in crop plants under stresses since gained knowledge could be applied to plants through genetic engineering that tackles the problem of abiotic stresses safeguarding. ABA-facilitated signaling pathways depend on the presence of at least one ABA-responsive element (ABRE) and either a coupling element or additional ABRE [55]. These major ABA signaling components actively regulate ABA communication pathways to address dehydration and other stresses. Huang et al. [99] studied the *BnbZIP3* gene that was upregulated under drought and salinity by exogenous application of ABA. The expression of *BnbZIP3* under different stresses showed that the promoter of this gene contained several *cis*-acting elements that were not only involved in ABA signaling but that this promoter was also simultaneously responsive to other stresses [99]. However, the promoters of other ABA-inducible genes do not contain ABRE *cis*-elements (Figure 3). Abe et al. [70] showed that the ABA-inducible RD22 promoter had no ABRE elements, even though this promoter could be strongly activated by ABA under various abiotic stresses. Gene-related studies indicated a correlation between ABA-dependent and ABA-independent pathways and cross-talks between players (ion pumps, Ca^2+^, ABA, TFs, mitogen-activated protein kinases, betaine, proline, reactive oxygen species, and DEAD-motif box helicases) that were involved in signaling pathways [62]. High throughput techniques identified other *cis*-elements that regulated ABA-induced gene expression. Yoshida et al. [91] investigated regulatory sequences such as G-box ABREs with the ACGTG core element that were recognized by bZIP TFs. More studies reported the involvements of AP2 (Apetala2)/ethylene responsive factor (ERF), myeloblastoma (MYB), No Apical Meristem (NAM)-Arabidopsis Transcription Activator Factor (ATAF)-Cup Shaped Cotyledon (CUC), collectively known as NAC, and zinc finger homeodomain (ZF-HD) TFs in ABA-induced gene expression (Figure 3) [100].

### 6.1. Basic Leucine Zipper (bZIP) Transcription Factors

**Figure 4:**
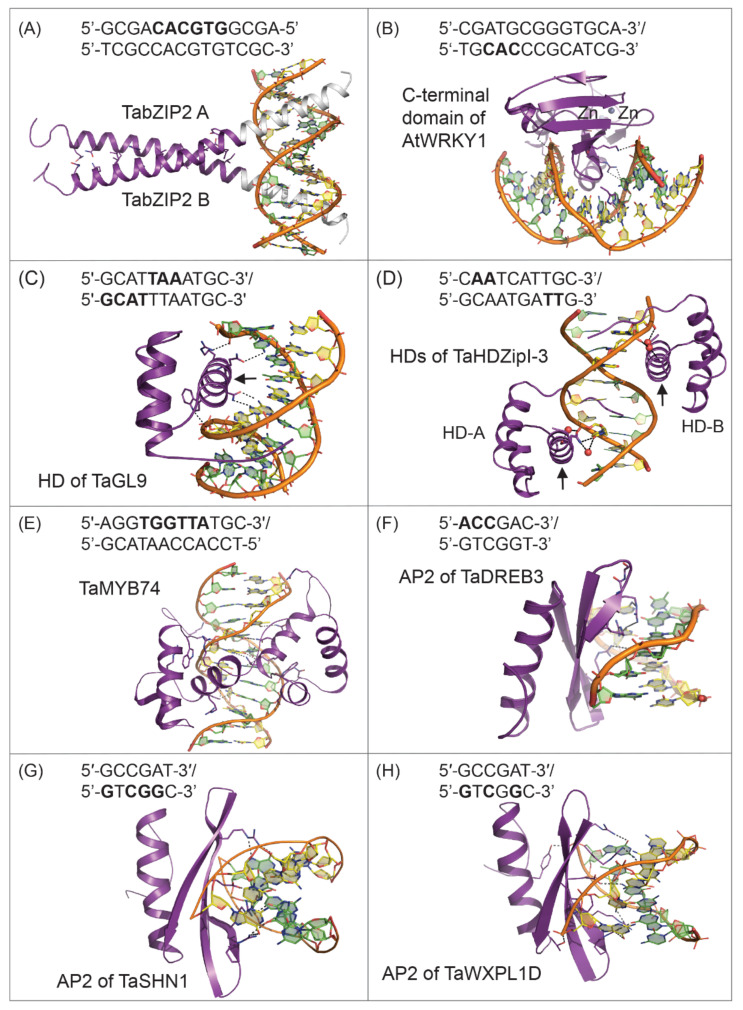
Structural characteristics of selected classes of wheat (*Triticum aestivum* L.) and *Arabidopsis thaliana* (L. Heynh) TFs involved in drought and other abiotic stresses. Coding and complementary DNA strands are shown in cpk yellow and cpk green sticks, respectively. Residues that interact with DNA are shown in cpk purple sticks and separations are illustrated in black dashed lines. DNA nucleotides (*cis*-elements) recognized by TFs are in bold. (**A**) bZIP: Molecular model of the wheat TabZIP2 homodimer (chains A and B) with the 5′-GGCCCACGTGGCCC-3′ ABRE *cis*-element. The transcription factor consists of basic (light grey) and leucine zipper (purple) regions (modified from [85]). (**B**) WRKY: Crystal structure of the C-terminal AtWRKY1 domain (PDB accession 1ODH) (traced in purple) illustrating antiparallel β-sheet contacting the 5′-CGATGCGGGTGCA-3′/5-TGCACCCGCATCG-3′) DNA fragment. Two zinc ions are shown in blue. Selected residues contacting DNA are shown at separations of ≤3.5 Å. (**C**) HD-Zip: Molecular model of the homeodomain (HD) of TaGL9 (purple) in complex with the 5′-GCATTAAATGC-3’/3’-GCATTTAATGC-5’ DNA fragment. The central recognition α-helix (perpendicular to the viewer; marked by an arrow) contains the residues contacting DNA at separations of ≤3.5 Å (modified from [137]). (**D**) HD-Zip: Molecular features of homo-dimeric TaHDZipI-3 homeodomains (HDs) (traced in purple) in complex with the 5′-CAATCATTGC/5′-GCAATGATTG-3′ *cis*-element. Mostly central recognition α-helices (perpendicular to the viewer; marked by arrows) carry residues that contact DNA through water (red spheres) mediated interactions at ≤3.4 Å separations (modified from [157]). (**E**) MYB: Molecular features of wheat TaMYB74 (traced in purple) in complex with the 5′-AGGTGGTTATGC-3′ MYBR1 *cis*-element. Predicted residues interacting with DNA at ≤3.6 Å separations are shown (modified from [26]). (**F**) DREB: Molecular model of the AP2 domain of TaDREB3 (traced in purple) in complex with the 5′-ACCGAC-3′/5′-GTCGGT-3′ DRE *cis*-element. Anti-parallel strands contact DNA at ≤3.4 separations (modified from [158]). (**G**) SHN: Molecular features of the wheat AP2 domain of TaSHN1 (traced in purple) in complex with the 5′-GCCGAT-3′/5′-GTCGGC-3′ CRT *cis*-element. Interactions between residues and DNA at ≤3.5 Å separations are shown (modified from [159]). (**H**) WXPL: Molecular features of wheat TaWXPL1D (traced in purple) in complex with the 5′-GCCGAT-3′/5′-GTCGGC-3′ CRT *cis*-element. Antiparallel strands carry residues that contact DNA at ≤3.5 Å separations (modified from [27]). Structural images were drawn in the PyMOL Molecular Graphics System v2.3.3 (Schrődinger LLC, Portland, OR, USA) using TF coordinates of 3D models computed and published in our works [26,27,85,137,157,158,159]. The coordinates of the AtWRKY1 domain were taken from the Protein Data Bank (https://www.rcsb.org/pdb, accessed on 14 April 2021).

The bZIP TFs make up one of the largest stress-inducible higher plant families that facilitate plant adaptation to stress conditions [55,73,93,101]. Crop plants contain different numbers of bZIP TF genes, e.g., 125 bZIP genes were reported to be regulated by abiotic stresses in maize, 92 in sorghum, 55 in tomato, 191 in wheat, 187 in wheat, 78 in *Arabidopsis thaliana*, 160 in soybean, 89 in rice, 63 in sesame, 114 in apple, 89 in barley, and 247 in rapeseed [73,102,103,104,105,106,107]. With the advancement of computer-based digital technologies, genome-wide analyses identified bZIP TFs in peanut, casava, and watermelon [84,108,109,110]. This information revealed that bZIP TFs mediated stress response in plants by the ABA-dependent pathway using specific ABRE *cis*-acting elements (ACGTG/TC to enhance the transcription of stress-responsive genes [6,42,84] (Figure 4). Based on conserved motifs, bZIP TFs are divided into A to S subfamilies, where for instance, subfamily A comprises ABA-responsive elements, named ABA-responsive element-binding proteins (AREB) or AREB-binding factors (ABF) that mediate stress signaling [56,91], while subfamily S contains bZIP TFs that respond to extreme stresses.

The bZIP TFs derive their name from a highly conserved bZIP domain consisting of a basic and a less conserved leucine zipper region. The bZIP region comprises 60–80 amino acid residues, whereas the basic region harbors a nuclear localization signal (NLS) followed by the conserved N-x7-R/K within the 18-residue motifs [56]. The leucine zipper region contains heptad repeats of leucine or other hydrophobic residues located exactly nine residues towards the COOH terminus, that are responsible for the dimerization of bZIPs. The basic region is present at the NH_2_-terminus and is responsible for sequence-specific DNA-binding activity and nuclear localization. In addition, bZIP can only bind the double-stranded DNA through the basic region after a dimer is formed. Das et al. [111] proved that the basic regions of bZIP (bZIP-bR) TFs are necessary and sufficient for DNA binding and DNA recognition specificity. Bioinformatic analyses and spectroscopic studies of bZIP-bRs suggested that monomeric bZIP-bRs could not bind and were disordered [111], meaning that bZIP TFs bind DNA as dimers, formed by the interaction of two α-helical stretches, consisting of seven residues per a DNA turn [84] (Figure 4A). Ellenberger et al. [112] revealed the two monomers bZIP-A and bZIP-B, interact through two monomeric α-helices with the pseudo-palindrome AATGACTCAT/TACTGAGTA sequence (Figure 4A).

In plants, bZIP TFs bind mainly to *cis*-elements with the ACGT core motif, such as A-box (TACGTA), C-box (GACGTC), and G-box (CACGTG) that induce the expression of downstream stress-responsive genes [56], thereby mediating various abiotic stresses including drought, salinity, and extreme temperatures [56,84,91,113]. The bZIP proteins were also reported to play vital roles in hormonal responses, biomass production, light signaling and photomorphogenesis, lateral root development, pollen germination, and seed maturation, flowering induction, and flower development [96,107,114]. The data defined synergistic interactions between specific members of distinct TF families, such as between bZIP and drought-response elements binding (DREB) TFs and between zinc finger-homeodomain (HD-ZF) and no apical meristem (NAM) *Arabidopsis* transcription activator factor (ATAF), and cup-shaped cotyledon (CUC)—the latter three groups collectively known as NAC are important in plant abiotic stress responses. DREB TFs also belong to ABA-independent dehydration-responsive TFs and not to the ABA-dependent dehydration-responsive TFs (Figure 4) [115], meaning that TFs of both pathways could interact under different abiotic stresses [6].

### 6.2. WRKY Transcription Factors

WRKY TFs, named after their WRKY-motifs, as one of the largest plant TF families play key roles in plant growth and development [95], and abiotic [69,113,116,117] and biotic [118,119,120] stresses. Reports showed that WRKY TFs play roles in both ABA-dependent and ABA-independent signal transduction pathways (Figure 4) under a variety of abiotic stresses [121]. Wang et al. [122] showed that three out of 15 WRKY genes in grapevines exhibited inducible transcription under ABA treatments. This suggested their participation in cold response via either ABA-dependent or ABA-independent pathways. It was demonstrated that WRKY TFs are regulators of ABA- and stress-related downstream genes such as *ABA1*, *ABA2*, *ABF2*, *ABF4*, *ABI1*, *ABI4*, *ABI5*, *MYB2*, *DREB1a*, *DREB2a*, *RAB18*, *RD29A*, and *cold regulated 47* [121], and that some WRKY TFs repressed the expression of ABA-responsive *ABI* (Arabidopsis ABA–insensitive) genes that contain APETALA2 domains [123]. The yeast one-hybrid screen of cDNA libraries prepared from plant roots under drought revealed 10 genes encoding wheat and maize WRKY TFs. These screens identified *Arabidopsis thaliana* WRKY TFs homologs, which were involved in ABA responses under stress [124], and several other genes of unknown function. Another important feature of WRKY TFs is that they regulate gene expression in diverse processes through the combination of positive and negative regulation [125]. WRKY13 overexpressing in *Arabidopsis thaliana* confers enhanced cadmium tolerance by binding to the PDR8 gene, which results in low Cd^2+^ accumulation [126]. In contrast, the overexpression of WRKY12 in *Arabidopsis thaliana* regulated Cd^2+^ tolerance negatively by repressing the glutamate-cysteine ligase (GSH1) gene expression [127]. Other features of WRKY TFs were also researched, such as their DNA *cis*-elements (Figure 4A) and downstream target genes [121]. Chen et al. [128] reviewed WRKY TFs in both model and crop plants and discussed high throughput genomic technologies in accelerating the translational research of WRKY.

High throughput genomic technologies and genome sequencing data from diverse plant species facilitated genome-wide computational analyses and identifications of WRKY families [129]. The members of the WRKY family include 74 genes in *Arabidopsis thaliana*, 107 in wheat, 81 in tomato, 197 in soybean, 70 in chickpea, 103 in rice, and 71 in pepper [95,117,129,130,131]. WRKY TFs form gene families with varying numbers, for example, 74 WRKY genes in *Arabidopsis thaliana* sub-classify in three groups based on the number of WRKY domains and patterns of Zn-finger-like motifs. Similarly, due to a large number and rapid expansion of the WRKY TFs gene family in plants, it was shown that monocotyledonous plants developed larger families than dicotyledonous plants [95] and that the rapid expansion of the WRKY TFs may be vital for adaptation and stress tolerance in monocotyledonous plants [95]. High throughput genomic and transcriptome analyses, gene chip analyses, real-time fluorescence quantitative PCR, and transgenic plants studied expression patterns and functional properties of WRKY TFs [122,132,133,134,135,136].

The 3D structure of the *Arabidopsis* WRKY COOH-terminal DNA-binding domain elucidated by X-ray crystallography (Protein Data Bank—PDB accession 2AYD) showed that it folds into a globular structure with five antiparallel β-strands and that the DNA-binding residues are positioned at β2 and β3 strands (Figure 4B). A Zn-binding site located at the end of β4 and β5 strands is crucial for shaping the overall structure and underlies the stability of WRKY. The WRKY DNA-binding domain consists of approximately 80 residues and contains the highly conserved WRKYGQK motif that is followed by Zn-finger-like binding Cys-Cys-His-His or Cys-Cys-His-Cys motifs located near the COOH-terminus of the DNA-binding domain [95]. The 3D structure of the *Arabidopsis thaliana* WRKY COOH-terminal domain suggested a potential mechanism for the transcriptional control and how the signal transduction events could be facilitated.

### 6.3. Homeodomain-Leucine Zipper (HD-Zip) Transcription Factors

HD-Zip TFs contain a highly conserved DNA-binding domain known as the homeodomain (HD) followed by a ZIP motif [137,138]. HD-Zip TFs have been classified in different families grounded on their features, such as the position of HD within sequences, associations with other domains, protein sizes, and a gene structure [137]. Based on the extensive analyses of HD-Zip TFs in higher plants, the HD-ZIP family is subdivided into four subfamilies (HD-Zip I to IV) [137]. Individual families can be distinguished by the conservation of HD-Zip domains additional conserved motifs, specific intron and exon distributions, structural features, and function. To date, 48 and 49 HD-ZIP genes were identified in *Arabidopsis thaliana* and *Oryza sativa*, respectively [139,140]. The phylogenetic analysis of rice and *Arabidopsis thaliana* genomes revealed specific subgroups of genes associated with each family. The isolation of the first HD-containing gene, maize *KNOTTED1* marked the discovery of several HD-ZIP TFs which were identified and characterized in model and crop plants through genome-wide analyses [141,142,143,144,145,146]. Further studies showed that HD-Zip TFs are actively involved in transcriptional regulation of abiotic stress responses, light, and hormonal (ABA, auxin, and ethylene) signal transduction, and plant growth and development [119,147,148,149,150,151,152,153,154,155,156]. Various members of subfamilies were identified with roles in developmental networks [6,152,155].

HD components of HD-Zip TFs are around 60 residues in length and consist of three α-helices (Figure 4C,D) connected by a loop and a turn [119,137,148,153]. The third α-helix, called the recognition α-helix (arrows in Figure 4C,D), is the most conserved across HD proteins and is responsible for high-affinity binding between HD-Zip TF and the major groove of the target DNA, while the disordered NH_2_-terminal component, located upstream of the first α-helix, interacts with the DNA minor groove [146]. During dimerization through monomeric α-helices, the HD-Zip structure forms a coiled-coil assembly (Figure 4C,D). The leucine zipper (LZ) of HD-Zip proteins is located downstream of HD and enables dimerization of HD-Zip TFs that is essential for DNA binding. Several HDs have a strong affinity to DNA as monomers, but HD-Zip TFs require dimeric assembly for an efficient DNA binding since monomeric HD-Zip TFs show a weak affinity for DNA binding.

Tron et al. [156] reported that HD-Zip I and II subfamily members share conserved HD and LZ domains, and bind pseudo-palindromic CAAT(A/T)ATTG or CAAT(C/G)ATTG *cis*-elements. In addition to HD and LZ domains, HD-ZIP II TFs contain a conserved CPSCE (Cys, Pro, Ser, Cys, and Glu) motif downstream of LZ [160], while members of HD-Zip III and HD-Zip IV subfamilies are identified by the presence of the steroidogenic acute regulatory protein-related lipid transfer (START) domain and a START adjacent domain (SAD) [161]. The presence of the COOH-terminal MEKHLA domain is a distinguishing feature of the HD-Zip III subfamily, which is absent in HD-ZIP IV TFs [135], and that the HD-Zip III subfamily members bind the consensus GTAAT(G/C) ATTAC sequence, while HD-Zip IV TFs bind TAAATG(C/T)A [162]. Tron et al. [156] showed that each HD of a dimer interacts with a different sequence, depending on the orientation relative to DNA, whereas only one HD of dimer contacts with a central nucleotide, and each monomer has a specific preference for the orientation of a *cis*-element. The key residues of HD domains that eventuate binding of a central nucleotide of *cis*-elements are conserved in each subfamily.

Mechanistic and functional aspects of how the HD-Zip TFs networks in plants sort out their roles in each subfamily during plant development and adaptation to abiotic stresses are not clear, and little is known on how downstream genes are regulated by HD-Zip TFs. The identification of downstream genes is important for building a comprehensive picture of the regulatory pathways, which will enable the validation of the suite of promoters controlled by HD-Zip TFs to define *cis*-elements. Once the potential downstream genes are defined, the promoter regions of known target genes can be investigated for the presence of specific *cis*-elements. This knowledge supports functional validation HD-Zip TFs and their cis-elements using high throughput techniques such as single base-pair mutations and deletions in combination with the promoter activation studies in transient expression assays.

### 6.4. Myeloblastoma (MYB) Transcription Factors

Myeloblastoma (MYB) TF family represents one of the frequent and functionally diverse classes of proteins found in plants. These TFs are characterized by the presence of highly conserved and variable numbers of MYB domain repeats at NH_2_-termini, which are involved in DNA-binding and protein-protein interactions. Conversely, the variable COOH-termini are responsible for controlling the regulatory activity of MYB proteins. Since the identification of the first MYB-like gene COLORED1 (CI) *from Zea mays*, which is essential for anthocyanin biosynthesis in the aleurone layer of maize kernels [163], many MYP TFs have been characterized. With the advancement in bioinformatics and genome sequencing resources, large MYB families have been described in several plant genomes, for example, in *Arabidopsis thaliana* (198), rice (183), and foxtail (209) [164]. MYB TFs have been shown to play crucial roles in regulating physiological, cellular, and biochemical processes, including plant development, cell cycle and morphogenesis, and plant responses to adverse stresses [165,166].

The classification of MYB TFs is based on the number of adjacent repeats in MYB domains. These were divided into four subfamilies including 1R-MYB, R2R3-Myb, 3R-MYB, and 4R-MYB with 1-4 repeats, respectively [150,163,165,167,168]. Each repeat consists of about 52 residues that fold into three α-helices (Figure 4E). The helix-turn-helix (HTH) structure is formed due to the second and third α-helices in each repeat within the domain [165,169]. The 3D structures of HTH revealed three tryptophan residues spaced regularly, resulting in a hydrophobic core reserved for DNA recognition and transactivation. It was reported that R2R3-MYB is the largest subfamily, abundant in both monocot and dicot plants with approximately 109 and 126 members in rice and *Arabidopsis thaliana*, respectively. R2R3-MYB TFs play roles in the regulation of multiple responses in plants, such as hormone signaling [67], regulation of differentiation, determination of cell shape, phenylpropanoid biosynthesis, and stress responses [127,166,170,171]. Based on the conservations of NH_2_-terminal DNA-binding domains and COOH-termini motifs of MYB domains, the members of R2R3-MYB TFs were classified into 23 subgroups [165].

Nuclear magnetic resonance (NMR) spectroscopy was used to reveal that R2 and R3 MYB domain repeats in viral MYB TFs are involved in target DNA recognition [172,173], and crystal structures of the mouse c-Myb (MsMyb), avian myeloblastosis virus (AMV) v-Myb, and *Trichomonas vaginalis* MYB3 (TvMyb3) revealed the involvement of a third α-helix of the R2 and R3 repeats in DNA recognition in the major groove [146]. Comparatively, plants contain far more MYB TFs than animal TFs, however, the structural information for plant MYB TFs is limited. Two studies reported the crystal structure of *Antirrhinum majus* RADIALIS (AmRAD), and *Arabidopsis* phosphate starvation response 1 (AtPHR1), showing that two copies of PHR1 MYB domains of 1R-type MYB TFs bind to the major groove of DNA [174]. However, despite the fact that this was the largest MYB TF group, no structures of plant R2R3-MYB proteins are known. Although many studies highlighted the biological function of R2R3-MYB TFs in plants, only recently, Wang et al. [175] elucidated the structure of R2R3-type MYB in *Arabidopsis* WER (AtMYB66). This analysis showed that the key residues in all R2R3-MYB proteins responsible for DNA recognition were highly conserved and that nearly half of R2R3-MYB members utilized the 5′-ACC-3′ sequence for DNA recognition, suggesting that these R2R3-MYB members may undergo subtle conformational changes or have other features that favor DNA binding.

### 6.5. Apetala2 (AP2)/Ethylene Responsive Factor (ERF) Transcription Factors

Several studies have focused on the ERF family of TFs through over-expression and transgenic plants. However, studies on these TFs with negative regulatory functions are few, although they could be employed in knockout mutational analysis in response to single or multiple stresses. Here, we summarize the mechanisms of the molecular basis of interactions and the role of recently studied ERF TFs in drought and associated stresses.

We focus on three classes of plant AP2/ERF TFs that have similar structural features and recognize similar *cis*-elements (Figure 4F–H). The AP2/ERF superfamily, firstly identified in *Arabidopsis thaliana*, represents one of the key plant-specific TF families [176]. This TF superfamily is characterized by the presence of at least one AP2 conserved domain consisting of approximately 60 residues [74,177]. AP2 and ERF domains share a sequence similarity that was first described in *Arabidopsis thaliana* and tobacco [177]. The AP2/ERF superfamily forms three families (ERF, AP2, RAV) and separate Soloist families whereby this subdivision is based on sequence similarity and the number of AP2/ERF domains [74]. The ERF family contains a single AP2 domain and forms two major subfamilies, ERF and DREB, based on sequence differences of their DNA-binding domains. The ERF/DREB subfamily contains stress-inducible TFs, which are involved in stress-induced gene expression in ABA-independent pathways [18]. Phylogenetic and domain analyses of the ERF family in *Arabidopsis thaliana* and rice divided these TFs into 12 and 15 groups, respectively and, it was emphasized that in addition to NH_2_-terminal DNA binding domains, COOH-terminal activation domains of AP2/ERF TFs mediate the activation of target gene expression [74]. Typically, the ERF subfamily members bind the ethylene-responsive element (ELE) with the core sequence AGCCGCC (GCC-box) [178] to confer biotic stress tolerance [158,178], while DREBs recognize the dehydration-responsive or C-repeat element (DRE/CRT) with the A/GCCGAC core sequence [158] (Figure 4F) to confer abiotic stress tolerance [179,180]. Structural comparisons of AP2 family TFs showed that β-sheets and α-helices locate to the NH_2_- and COOH-terminal parts of domains, respectively [158]. Some members of this family have a pair of AP2 domains that bind GCAC on stress-responsive gene A/G)N(A/T)TCCC(A/G)ANG(C/T) sequences [181].

Further evidence revealed that the members of the AP2/ERFs family are key players in diverse biological and developmental processes and regulate genes by transcriptional and post-translational regulatory mechanisms [179,182], and signaling networks [183]. The DREB subfamily members are the main regulators of various abiotic stresses [184], but also affect plant developmental processes [185]. The RAV family members possess a single AP2 domain, and another conserved DNA-binding domain is known as B3, which binds to CAACA and CACCTG sequences at the COOH-terminus, these TFs also play roles in plant development and stress responses [186]. The Soloist TF families usually contain a single AP2 domain but display more diverse sequences compared to ERF, AP2, and RAV families. Soloist TFs have a unique position in the AP2/ERF superfamily and were suggested to be involved in SA accumulation, especially in defense against bacterial pathogens [187]. AP2/ERF TFs were studied using the gene-editing technology [188], which helped explore the function of AP2/ERF TFs [189], and in biotechnological applications of AP2/ERF TFs to crops [18,158,188,190,191,192].

Recent developments in high throughput gene technologies made accessible complete genome sequences and facilitated genome-wide identification of AP2/ERF superfamily members in *Arabidopsis thaliana*, wheat, soybean, rice, barley, cotton, grape, sorghum, alfalfa, poplar, tomato, Chinese cabbage, pearl millet, peach, cucumber, and peanut [74,193,194,195,196,197]. Computational analyses and expression studies of the AP2/ERF superfamily indicated that it plays an important role in plant growth and development [196].

#### 6.5.1. Drought-Response Elements Binding (DREB)/C-Repeat Binding Factor (CBF) Transcription Factors

DREB/CBF TFs include plant-specific TFs that bind the DRE/CRT *cis*-element in the promoter region of a stress-responsive gene [42]. DREB/CBF TFs play an important role in ABA-independent pathways, regulating a series of downstream gene (which contain DRE/CRT elements) expressions in response to various abiotic stresses such as drought, cold, salt, heat, etc. [179,198,199,200]. DREB TFs constitute a large part of the AP2/ERF superfamily, which was first reported in *Arabidopsis thaliana* [176,201]. Based on structural features, the DREB subfamily is divided into six groups A1-A6, where A1 and A2 constitute the two largest groups [158,202,203,204]. The characteristic feature of DREB proteins is the presence of the 60–70 residue-long AP2 domain, without other obvious homology in the sequences of other regions [201]. Chen et al. [205] described seven residues in the AP2 domain that play a key role in the DRE/CRT element-binding and these include four Arg, two Trp, and one Val residue. Furthermore, the 14th Val residue is a characteristic feature of the subfamily and is the main site of interactions with DNA. The 3D analysis of the AP2/ERF domain showed three-stranded β sheets, connected by anti-parallel α-helical loops, which are packed analogously to each other [206] (Figure 4F–H), and the AP2 domain was divided into YRG and RAYD regions. YRG consists of 20 residues that are rich in basic and hydrophobic residues at each NH_2_-terminal stretch, which led to the suggestion that YRG is involved in the DNA-binding function. Conversely, RAYD contains 40 residues, where the RAYD motif has a conserved core region that can form an amphipathic α-helix in the AP2 domain—this structural element was suggested to participate in protein-protein interactions. Differences in AP2 domains of the DREB subfamily and other subfamilies occur at specific motif sites that include Val14 and Glu19 residues, which are conserved. Mizoi et al. [179] and Amalraj et al. [158] highlighted major functions of DREB TFs in a variety of abiotic stresses.

Wang et al. [204] described that the whole-genome duplication has played important roles in the expansion of the DREB family in plants. The complete genome sequences of both model and crop plants are available, which allowed detailed functional analyses of ERF and DREB genes in *Arabidopsis thaliana*, *Glycine max*, *Zea mays*, *Triticum aestivum*, *Brassica rapa*, *Brassica oleracea*, *Brassica napus*, *Vitis vinifera*, *Malus domestica*, *Oryza sativa*, *Phaseolus vulgaris*, and many more plant species [74,158,207,208,209,210,211,212,213,214]. Although the distribution of DREB TFs in different plants varies, studies reported the occurrence of multiple genes in plants, for example, 30 DREB genes in *Morus notabilis* [215], 41 in *Hordeum vulgare* [197], 81 and 99 in *Musa acuminata* (A genome) and *Musa balbisiana* (B genome), respectively [202], 79 in *Zoysia grass* [216], 39 in Jujube [217], and 30 in *Vigna radiata* [218].

#### 6.5.2. Shine (SHN) Transcription Factors

The AP2/ERF TF superfamily mostly contains plant-specific TFs involved in various processes during growth and development, including wax biosynthetic pathways [74,219,220,221]. Several studies highlighted the significance of the cuticular wax layer that covers the aerial epidermis of plants since this extracellular hydrophobic cuticle enhances drought tolerance by reducing a nonstomatal water loss and provides protection against various biotic and abiotic stresses [220,221]. Bernard and Joubes [222] and Borisjuk et al. [220] explained the chemical composition of the cuticle, which consists of a cutin polyester matrix covered with epicuticular waxes and filled with intra-cuticular waxes that enhances drought tolerance by preventing water loss from transpiring leaves. Further, Oshima et al. [223] in *Arabidopsis thaliana* and Borisjuk et al. [220] in maize revealed genes involved in plant wax biosynthesis.

The SHINE (SHN/WIN) clade TFs belong to the AP2/ERF superfamily (Figure 4G) and were first reported in *Arabidopsis thaliana* as transcriptional activators playing roles in the regulation of the cuticular wax biosynthesis [224], tomato [225], rice [226], apple [227], and wheat [159,228]. Further reports suggested that WIN1/SHN1 activated the expression of genes involved in cutin biosynthesis and indirectly affected cuticular wax production [229]. Wax biosynthesis is one of the complex pathways and the coordinated expression of multiple related genes is required for the biosynthesis of cuticular waxes [230]. These multiple genes are transcriptionally regulated by several TFs through specific recognition of *cis*- and *trans*-acting factors [229,231,232]. Several TFs, including those encoded by the *Arabidopsis* SHN/WIN family and alfalfa (WXP1 and [220,233] were reported to regulate cutin or cuticular wax biosynthesis.

The WIN1/SHN1, a small protein with 199 residues was reported to be involved in several developmental processes including cutin biosynthesis and ethylene-mediated signaling, and wax metabolism. The SHN/WIN subfamily has 12 members, however, not all display the glossy leaf appearance. Plants overexpressing the *Arabidopsis thaliana* SHN/WIN genes mediated wax accumulation leading to a glossy phenotype [220] and resulted in upregulation of other related genes involved in wax biosynthetic pathways [234]. Shi et al. [229] demonstrated that silencing of SHN/WIN clade genes negatively impacted many plant characteristics such as flower cutin biosynthesis, and modified petal cell wall structure which altered floral organ morphology. Sela et al. [235] found that AtSHN1 conferred drought tolerance and improved defense resistance when overexpressed, while the SlSHN1 gene overexpressed in tomato, exhibited improved drought tolerance due to the enhanced cuticular wax accumulation [231]. These findings indicated that SHN/WIN TFs are responsible for the regulation and accumulation of cuticular wax in plants leading to enhanced drought tolerance by decreasing nonstomatal water loss [223,224].

#### 6.5.3. Wax Production-Like (WXPL) Transcription Factors

The wax production WXP1 and WXP2 TFs identified in model legume *Medicago truncatula* are the members of the AP2/ERF superfamily, and earlier studies reported their involvement in cuticle biosynthesis regulation in plants [219,233]. The structure of the WPX1 DNA-binding domain and its high level of protein sequence identity (53%) with RAP2.4 suggested that WPX1 is homologous to ERF TFs [27,220]. Conversely, other reports indicated that a low level of protein sequence identity existed within the members of the WIN1/SHN1 clade of the AP2/ERF family [219], which represented a clade of intensely studied TFs involved in transcriptional regulation of cuticular wax biosynthesis and its distribution in aerial parts of plants [27,220,225,229,235,236,237]. Zhang et al. [219] reported that both WXP genes show ABA- and abiotic stresses-dependent expressions. The direct evidence of WPX involvement in several abiotic stresses came from their orthologues. Amongst WXP proteins was *Arabidopsis* RAP2.4, which was involved in light and ethylene signaling whereby the light stress downregulated while drought and salt stresses upregulated its expression [238]. The ZmDBF1 protein is another WXP orthologue, which was initially induced by ABA and abiotic stresses such as drought and drove the expression of drought inducible ZmRab17. Similarly, BpDREB2 from *Broussonetia papyrifera* was identified as an orthologue of WXP2 that had three characteristic domains/motifs: AP2, a nuclear localization signal, and a COOH-terminal acidic activation domain. Bi et al. [27] identified molecular features of wheat WXPL in complex with DNA that bound CRT, DRE, and GCC *cis*-elements (Figure 4H). Similar to ZmDBF1, this TF also showed a specific binding affinity to DRE via a yeast-one-hybrid assay [198]. Similarly, another *GhDBP2* WXP2-like gene in cotton, encoding a DREB (A-6) subfamily protein was reported that was induced by the ABA treatment in cotyledons and abiotic stresses drought, high salinity, and low temperature.

Numerous findings showed a nearly 40% increase in a total leaf wax content per unit surface area by constitutively over-expressing WXP1 in transgenic *Medicago sativa* [219]. Similarly, the expression of WXP1 and its WXP2 homologue in transgenic *Arabidopsis thaliana* led to significantly higher cuticular wax deposition on leaves, which was detected visually [219,233]. Transgenic *Arabidopsis thaliana* and alfalfa plants had also improved physiological performance and enhanced drought tolerance [219,233]. Surprisingly, detached leaves of both WXP1 and WXP2 transgenic plants retained more water than control leaves from wild-type plants and both transgenic plants showed significantly enhanced survival under drought. By contrast, WXP1 expressed in *Arabidopsis thaliana* caused improved tolerance to freezing temperatures, while plants expressing WXP2 were more sensitive to low temperatures compared to control plants [233], although a direct connection between changes in cuticle properties and freezing tolerance was not shown. Additionally, the expression of WXP1 in alfalfa and WXP2 in *Arabidopsis thaliana* strongly interfered with the growth and development of transgenic plants. The latter authors reported that the expression of WXP1 in transgenic *Arabidopsis thaliana* showed no negative effects on plant development while transgenic plants expressing WXP2 showed reduced growth [219,233]. Bi et al. [27] isolated WXP-like transcripts from drought-tolerant wheat and suggested the role for wheat WXP-like TFs in the regulation of cuticle wax biosynthesis—these authors proposed that the roles for WXPL and cuticle-related MYB TFs in the regulation of genes responsible for the synthesis of cuticle components are linked (Figure 2A) [27].

The implication of ERF proteins (and other TFs) in abiotic stress tolerance is due to the presence of functionally conserved domains as target markers for crop improvement upon abiotic stress tolerance through genetic modification [207]. Several studies have shown that identification and isolation of such DNA binding factors may reveal novel regulation patterns under stress conditions [239,240].

## 7. Natural Variations in Transcription Factors during Drought and Associated Stresses

There is evidence that abiotic stresses could simultaneously affect plant growth and development [14,241,242,243,244,245,246,247], and that plants evolved sophisticated mechanisms to withstand multiple abiotic stresses due to strong selection to adapt to prevailing conditions [2,23,248,249,250]. Addressing multiple stresses by planned experimentation is a major challenge due to the complexity of exposure to these multiple stresses. To gain an insight into the plant adaptation to various stress-inducing conditions, both natural variation and complex mechanisms underlying stress tolerance/resistance should be considered [86,243,247]. Exploring natural variations contributed significantly towards elucidating the gene function without the confounding effects of expression outside of the natural genomic context [250]. Studies in natural variation provided novel insights into adaptive mechanisms shaping plant stress responses and helped uncover novel loci involved in stress responses [245]. For example, drought-responsive genes showed natural variation and allelic variation on previously described loci and novel loci [251,252]. Rao et al. [251] uncovered two alleles for DREBA1 in *Solanum pimpinellifolium*, using a screen of 94 genotypes. These alleles together accounted for 25% of trait-associated phenotypic variation [253]. Additional studies of allelic variation in TFs and downstream drought-responsive genes significantly contributed to plant selection and adaptation [165]. Here, GsZFP1, a new C2H2-type zinc-finger TF, was identified in the soybean wild relative *Glycine soja* [254] and the overexpression of GsZFP1 in alfalfa led to the high expression of various drought-responsive genes [82]. Similarly, Arms et al. [253] mapped a QTL in *Solanum habrochaites*, a drought-tolerant wild tomato that co-localized to C2H2-type zinc-finger TFs on chromosome 9 of the cultivated tomato. Natural variations were explored in the CBF gene family of complex evolutionary patterns. The CBF regulon consists of three regulatory proteins CBF1, CBF 2, and CBF 3, which play key roles under freezing stress. This was supported by population-level investigations in wild tomato *S. peruvianum* and *S. chilense*. Mboup et al. [254] found that CBF3 showed a reduced nucleotide diversity across all populations/species consistent with the strong purifying selection at that locus. Mboup et al. [254] using population-level data also highlighted the complex evolutionary history of *CBF* genes and showed the advantage of using natural variation to uncover the gene function within a genomic context.

Currently, phenotyping platforms can be used for screening thousands as opposed to hundreds of individual plants for tolerance traits [255,256,257,258,259]. Natural variation can also be used for understanding the genetic architecture of complex traits such as plant tolerance to stresses. For example, association studies in combination with the genetic disequilibrium linkage, are contributing towards dissecting complex trait loci in plants [260]. This technique highlights more precisely the resolution of genome-wide association studies (GWAS) at the gene level, subject to the availability of high-density and genome-wide DNA markers [261]. Additionally, after genome-scale sequencings of large numbers of varieties with different genetic backgrounds are available, GWAS accelerates the genetic dissection of complex traits in crops using natural variations [47,261]. Similarly, Yan et al. [259] suggested the candidate gene association analysis as one of the methods of choice for the discovery and detection of single nucleotide polymorphism. This technique ensures that markers are within or closely linked to genes that contribute to complex traits [259].

## 8. Conclusions and Outlook

In this review, we examined the aspects of plant tolerance to extreme abiotic stresses such as drought and how stress-inducible TFs participate in drought, which in turn regulate the expression of a large array of downstream genes. We advocate that TFs are powerful tools for genetic engineering as their controlled expression can lead to the up- or downregulation of genes under their control. The discovery and descriptions of structure-function relationships of several classes of plant TFs aided in the identification of pathways that control the plasticity of plant growth and control the modulation of plant development in response to abiotic stress. However, the work on plant defense mechanisms controlled by TFs is ongoing. While we need to acknowledge that the progress on molecular mechanisms of TFs progresses, in the future we need to provide precise molecular descriptions of the function of TFs to understand their precise biological roles. It will be critical to describe: (i) Molecular basis of formation of transcriptional activation and repression complexes, and the influence of post-translational modifications on the formation of these complexes; (ii) mechanisms of activation and repression of target genes, of how TFs form oligomeric assemblies; (iii) 3D structures and folding pathways of TFs, and how structural determinants play roles in DNA recognition and in the activation of complexes; (iv) mechanisms on how these complexes are regulated. Answers to these questions will allow us to develop modified versions of TFs with improved DNA-binding properties and create TFs that will be independent of other upstream regulatory pathways. This information will be useful to define the mechanisms of formation of functional complexes of TFs, and most importantly, the new knowledge could hold promises for informed decisions on suitable TF applications to bioengineer plants with enhanced tolerance to drought and other abiotic stresses.

## Data Availability

Not applicable.
